# Impact of Updating Pharmacogenetic Results: Lessons Learned from the PREDICT Program

**DOI:** 10.3390/jpm11111051

**Published:** 2021-10-20

**Authors:** Michelle Liu, Sara L. Van Driest, Cindy L. Vnencak-Jones, Leigh Ann G. Saucier, Bartholomew P. Roland, Cheryl L. Gatto, Shari L. Just, Asli O. Weitkamp, Josh F. Peterson

**Affiliations:** 1 Department of Pharmacy, Vanderbilt University Medical Center, Nashville, TN 37232, USA; 2 Department of Medicine, Vanderbilt University Medical Center, Nashville, TN 37232, USA; sara.van.driest@vumc.org (S.L.V.D.); josh.peterson@vumc.org (J.F.P.); 3 Department of Pediatrics, Vanderbilt University Medical Center, Nashville, TN 37232, USA; cindy.vnencak-jones@vumc.org; 4 Department of Pathology, Microbiology, and Immunology, Vanderbilt University Medical Center, Nashville, TN 37232, USA; 5 Vanderbilt Institute for Clinical & Translational Research, Vanderbilt University Medical Center, Nashville, TN 37232, USA; leighann.saucier@vumc.org (L.A.G.S.); broland@abalonebio.com (B.P.R.); cheryl.l.gatto.1@vumc.org (C.L.G.); 6 Department of Biostatistics, Vanderbilt University Medical Center, Nashville, TN 37232, USA; 7 Health IT Decision Support and Knowledge Engineering, Vanderbilt University Medical Center, Nashville, TN 37232, USA; shari.l.just@vumc.org; 8 Department of Biomedical Informatics, Vanderbilt University Medical Center, Nashville, TN 37232, USA; asli.ozdas@vumc.org

**Keywords:** pharmacogenomics, pharmacogenetics, personalized medicine, precision medicine, SSRIs, reprocessing, reinterpretation

## Abstract

Pharmacogenomic (PGx) evidence for selective serotonin reuptake inhibitors (SSRIs) continues to evolve. For sites offering testing, maintaining up-to-date interpretations and implementing new clinical decision support (CDS) driven by existing results creates practical and technical challenges. Vanderbilt University Medical Center initiated panel testing in 2010, added *CYP2D6* testing in 2017, and released CDS for SSRIs in 2020. We systematically reinterpreted historic *CYP2C19* and *CYP2D6* genotypes to update phenotypes to current nomenclature and to launch provider CDS and patient-oriented content for SSRIs. Chart review was conducted to identify and recontact providers caring for patients with current SSRI therapy and new actionable recommendations. A total of 15,619 patients’ PGx results were reprocessed. Of the non-deceased patients reprocessed, 21% (*n* = 3278) resulted in CYP2C19*1/*17 reinterpretations. Among 289 patients with an actionable recommendation and SSRI medication prescription, 31.8% (*n* = 92) did not necessitate contact of a clinician, while 43.2% (*n* = 125) resulted in clinician contacted, and for 25% (*n* = 72) no appropriate clinician was able to be identified. Maintenance of up-to-date interpretations and recommendations for PGx results over the lifetime of a patient requires continuous effort. Reprocessing is a key strategy for maintenance and expansion of PGx content to be periodically considered and implemented.

## 1. Introduction

The evidence for translating pharmacogenomic (PGx) results to practice is evolving, expanding, and increasingly formalized into clinical guidelines that are periodically updated [1]. Successful implementation of panel-based PGx testing, driving automated clinical decision support (CDS), has now been described at several institutions [2,3,4,5,6,7,8,9]. For automated guidance to stay relevant, these programs must develop and implement a strategy to update interpretations and clinical guidance for historical genetic results. The challenges faced by these programs are substantial, as standard laboratory information systems and electronic health records (EHRs) are not typically designed to manage reinterpretation or expansion of genetic results [9,10]. In this manuscript, we describe the process of revising historical PGx to support a longstanding clinical PGx program within an academic medical center. We focus on the impact of updating *CYP2C19* and *CYP2D6* interpretations and the downstream impact on CDS for selective serotonin reuptake inhibitors (SSRIs), as the majority of our tested patients have *CYP2C19* results.

## 2. Background

### 2.1. Setting

Vanderbilt University Medical Center (VUMC) implemented PGx testing with the Pharmacogenomic Resource for Enhanced Decisions in Care and Treatment (PREDICT) initiative in 2010 [11]. A primary goal of PREDICT is to provide clinical guidance for PGx results through an automated workflow tied to the laboratory information system, EHR, and patient health record [12]. From the start of the program, PREDICT implemented panel-based PGx testing; however, interpretation was initially limited to single PGx gene effects on a single medication (e.g., *CYP2C19* and clopidogrel for cardiovascular indications). Subsequently, the program expanded to include more drugs, more genes, and complex PGx scenarios, such as: (1) addition of drug-specific CDS associated with multiple genes, including warfarin (based on *CYP2C9* and *VKORC1)* and (2) addition of PGx-tailored drug dosing with multiple indications, such as for voriconazole and ondansetron [12]. The evidence for each drug–gene interaction is reviewed prior to its incorporation into clinician-facing and patient-facing electronic health portals. As the program has matured, the institutional standard for interpretation and clinical guidance has adhered closely to the Clinical Pharmacogenetics Implementation Consortium (CPIC) guidelines [13]. These guidelines, by design, are periodically updated to incorporate new evidence. For example, before 2016, CPIC’s guidelines for clopidogrel, tricyclic antidepressants, and SSRIs defined four CYP2C19 phenotype groups (poor, intermediate, extensive, and ultrarapid metabolizers), whereas guidelines and updates published after 2016, including those for voriconazole and proton pump inhibitors, defined five phenotype groups (differentiating rapid from ultrarapid metabolizers), consistent with term standardization efforts [14,15]. In addition, *CYP2D6* activity score ranges have been redefined for phenotype definitions [15,16]. At VUMC, we sought to redefine PGx interpretations and update CDS by reprocessing our historical patient results. 

### 2.2. Definitions

For the purposes of this manuscript, PGx genotypes are referred to as results while PGx phenotypes (e.g., metabolizer status) are referred to as interpretations. Guidance suggesting an alternative drug, dose adjustment, or consultation is referred to as recommendations. Redefining or updating an interpretation based on new guidance, including updated results for standardization in nomenclature, is referred to as reinterpretation. Meanwhile, the process of systematic reinterpretation is referred to as reprocessing. Actionable PGx interpretations are those that trigger drug-specific CDS in our local EHR [12]. Reinterpretation for the purposes of this manuscript does not entail reanalysis or retesting of patient DNA. Rather, reinterpretation refers to the process of applying new scientific knowledge to (unchanged) PGx test results to produce a standardized or remapped phenotype. 

### 2.3. Objective

**Figure 1:**
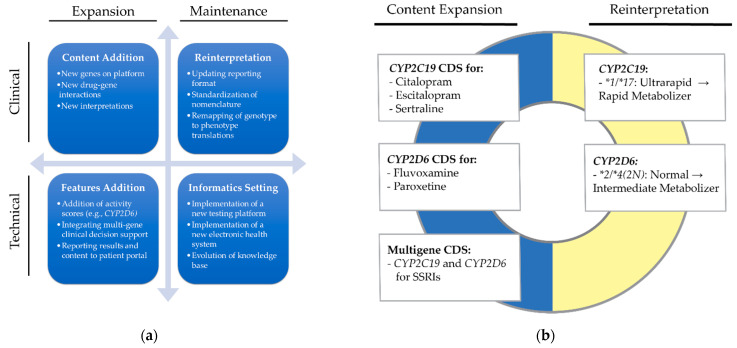
Potential utility of reprocessing pharmacogenomics results. (**a**) Different types of reprocessing utility; (**b**) specific use cases of reprocessing to support clinical maintenance and SSRI content expansion (example historic results and reinterpreted phenotypes are listed).

Reprocessing is a strategy that can accomplish two objectives for PGx programs—maintenance of the PGx content and content expansion (Figure 1a,b). An automated method of reprocessing ensures consistency and may save time and effort compared to manual processes when there are a large number of results to be reprocessed. Reprocessing for maintenance includes updating for the most current PGx nomenclature and incorporating any codified modifications to expert and regulatory guidelines. Reprocessing for content expansion maximizes clinical utility of historic results and clinical support for additions to the content of a genetic panel. Reprocessing may also be necessary due to technical advancements such as the transition to a new PGx assay, new EHR, and/or a more robust knowledge database (Figure 1a). For example, a switch in testing assay could require reprocessing to normalize the interpretation and nomenclature that emerged between the old and the new assay and associated testing platforms. Knowledge database updates can also trigger reprocessing; for example, adding *CYP2D6* activity scores to the knowledge base requires reprocessing so the activity scores can be utilized by CDS. In each case, reprocessing may be required to harmonize terminology, results, and interpretations to support recommendations after implementation of the new technology. At VUMC, reprocessing was necessary to update *CYP2C19* and *CYP2D6* interpretations and support new multigene SSRI CDS leveraging previously reported results (Figure 1b) [15,16]. We reprocessed and reinterpreted several pharmacogenes, but our focus in this manuscript will be on *CYP2C19* reinterpretations and the impact of adding CDS for SSRIs [17]. Reinterpretations for *CYP2D6* based on 2019 term standardization is planned to occur in the near future [16]. Based on our experiences of reprocessing PGx results within a large academic medical center, we share the impact, highlight the lessons learned, and provide guidance on one strategy to maintain updated PGx interpretations and expand CDS that will benefit both new and established PGx programs alike. Our focus is on clinical PGx results.

## 3. Materials and Methods

### 3.1. Environment

While there is no one-size-fits-all implementation approach to PGx, we highlight some of the key environmental components that have allowed reprocessing at our institution [12]. Access to discrete results is essential to reprocessing efforts. Without discrete results, such as results scanned into the EHR, automated reprocessing is difficult or impossible to accomplish. We leverage in-house testing and an integrated informatics infrastructure to enable storage of discrete results in an accessible database since 2010. A second environmental component is personnel with clinical knowledge. Clinical expertise is necessary to (1) guide remapping for reinterpretation, (2) interpret clinical guidelines and recommendations, and (3) to provide clinical support to clinicians relating to reinterpretations. The third component is an analytic pipeline infrastructure. This type of information technology is required for automated reprocessing of clinical content updates. In addition to these key components, clinical needs and institutional initiatives drive the reprocessing effort forward, and collaboration with institutional oversight (e.g., Pharmacy and Therapeutics committee) can ensure proper prioritization, integration, and execution. 

### 3.2. Initiation of Reprocessing

An institutional opportunity cost was evaluated prior to the decision to initiate new SSRI CDS and reprocessing. This was done by reviewing the institutional inpatient and outpatient SSRI prescription rates, psychiatric patient volume, as well as potential benefits derived from providing new CDS based on old results. Clinical needs and patient impact, such as the SSRI prescription volume, supported the decision to implement SSRI CDS. Program leadership secured institutional approvals for initiation of new CDS implementation, reprocessing decisions, and resource allocation. 

### 3.3. Automated Processes

In a previous report, we describe the informatics infrastructure and the data flows enabling the program’s automated CDS [12]. As described above, there are two major use cases that necessitate reprocessing of PGx results: (1) new PGx interactions, and (2) changes to existing PGx interpretations. An automated analytics pipeline to support reprocessing is a key component of a successful PGx program, enabling sustainable CDS. The process is initiated by identification of all eligible patients impacted by the interpretation changes. The PGx results of these patients are subsequently processed by referencing the knowledge base updates to compute reinterpretations. In our EHR system (EPIC), this automated process enables propagation of necessary reinterpretations into the identified patients’ Genomic Indicators in the EHR driving CDS. In addition to the automated pipeline, reprocessing also includes ad hoc reporting to identify eligible patients who are already on the medications that trigger CDS, requiring an evaluation for the applicability of the PGx interpretation changes on these patients. This assessment is utilized to notify providers with patients who may benefit from a change in their prescriptions and is discussed further below. Overall, by managing the results in relational databases and engaging the data analytics team to assess longitudinal impact, the programmatic footprints are preserved to enable reprocessing.

### 3.4. Manual Processes

Development of new SSRI CDS and reinterpretation was guided by CPIC guidelines and *CYP2C19* term standardization [15,17]. The new content included SSRI CDS in the form of best practice alerts (BPAs), Genomic Indicators, and patient-facing interpretations accessible in the patient portal, My Health at Vanderbilt (MHAV). Following this development, new patients received the entire suite of variants from the genotyping platform and associated CDS. However, the historical results were largely produced by a previous genotyping platform with different *CYP2C19* variants and without *CYP2D6* variants or a copy number assay. In order to release CDS for historically tested patients, clinical judgement and documentation of transitions became imperative to accurately standardize phenotype terms, reinterpret prior results, and to determine if retesting was required. 

**Figure 2:**
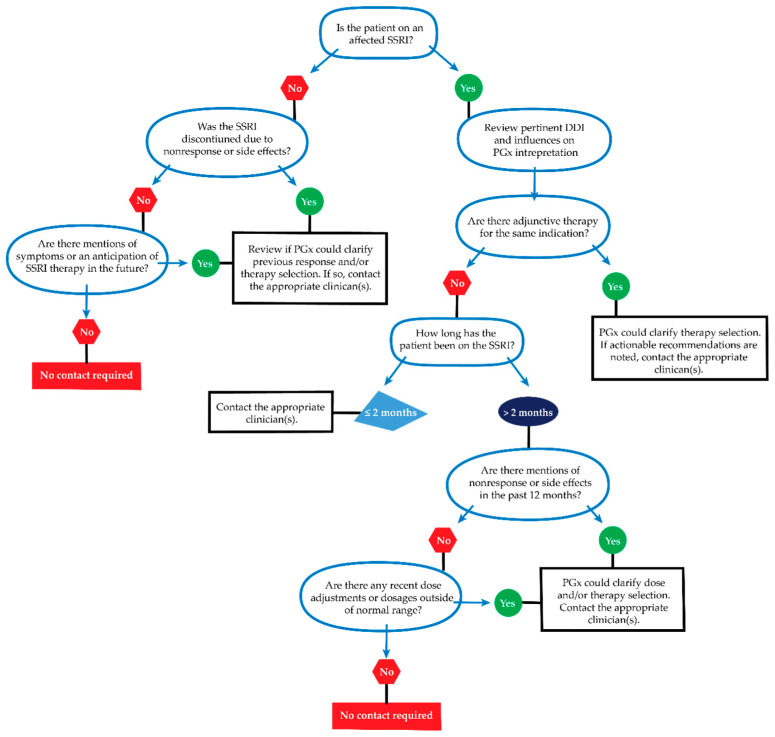
Clinician contact decision tree for actionable SSRI reinterpretations. The programmatic reprocessing effort flagged patient records with evidence of new actionable reinterpretations and SSRI prescription. Patient records were reviewed using this workflow to determine the appropriateness of clinician contact.

We anticipated that reprocessing could clinically impact patients without prior actionable interpretations and recommendations for SSRIs. Therefore, a manual process was in place to review patient charts and contact clinicians for those patients with new actionable recommendations (Figure 2). The PGx results were available prior to reprocessing and the SSRI recommendations and BPA were available immediately after reprocessing in the EHR, thus we did not send alerts about reprocessed results to all clinicians, as this may contribute to alert fatigue. Given the outpatient and chronic nature of SSRI therapy, some clinicians may not see the patient and relevant BPAs for several months. Thus, we did not want to rely solely on the BPA alerts (which fire on generation of a new prescription or medication order for a patient with an actionable PGx result). We used a manual process of clinician contact to mitigate the delay in automated communication of recommendations while avoiding mass alerts for updates irrelevant to a patient’s care.

Our criteria for recontacting clinicians were designed to be broad enough to ensure that we did not unintentionally overlook any potential patients with actionable reinterpretations. The criteria for recontact included non-deceased and active patients currently on a PGx-relevant medication with a nonactionable to actionable reinterpretation transition (most commonly no prior SSRI recommendation to an actionable SSRI recommendation). Active patients were defined as those individuals interacting with our healthcare system within the last two years. Once a patient had met these criteria, an appropriate clinician was identified through review of notes and encounters. If no primary care or psychiatry specialist could be identified, then the most recent or most suitable clinician was contacted. Templated language was drafted to include an explanation of the program’s reprocessing goal, reinterpretation, and relevant recommendations; however, relevant patient-specific information was also included in the message to better inform and tailor guidance for clinicians (Figure S1). A clinical pharmacist was available to further consult on any additional questions. 

### 3.5. Organization Resources and Governance

Maintenance and expansion of a PGx program is a multidisciplinary team effort [12]. Here, we outline the team members and their involvement in reprocessing. Although some core members have been involved in all aspects of the PGx program, most of the team members involved in the reprocessing efforts have additional responsibilities in the institution and are not specifically dedicated to the PGx program. 

Clinical subject matter experts (SMEs) and the molecular diagnostics laboratory director defined the results for reinterpretation and standardization. The molecular diagnostics laboratory updated the laboratory report to include current nomenclature for variants associated with SSRI interpretations. The SMEs created CDS content for SSRI BPAs, Genomic Indicators, and patient interpretations prior to reprocessing. The CDS content was reviewed by the Pharmacy and Therapeutics committee as well as the relevant subcommittees, while the patient-facing content in MHAV was reviewed by Patient Education. During the reprocessing effort, the SMEs determined which reinterpretation was considered clinically actionable, and they acted as coordinators of care to ensure a clinician was aware of any updated recommendations after reprocessing. Chart review was conducted for patients flagged for actionable PGx reinterpretations, and a message was sent to the treating clinician(s) if a patient’s reprocessed results changed from nonactionable (or absent) to actionable. Questions and concerns from clinicians and patients regarding reprocessing and reinterpretations were triaged by programmatic staff and then addressed by clinical SMEs.

Health bioinformaticians updated the integration architecture comprised of the knowledge base and the corresponding translational rules engine to facilitate multigene support for five new SSRI DGIs. Reprocessing was facilitated by the bioinformaticians that required quality and control testing prior to releasing the updates. 

### 3.6. Data Collection

Data were collected retrospectively after the reprocessing effort in 2020. Data were sourced from operational reports, dashboards, and databases linked to the electronic health system used for the reprocessing initiative (e.g., Clarity, Tableau).

## 4. Results

### 4.1. Reprocessing Timeline

The reprocessing effort took over 1 year of planning and preparation and 2.5 months of pre-implementation work. This included building the necessary technical components, running historic results through a translational engine, and finally multiple rounds of validation in different testing environments to ensure no issues are identified. Once validation was complete, the build was implemented for release into the EHR environment, and the subsequent validation processes were repeated. 

### 4.2. Patient Cohort

**Figure 3:**
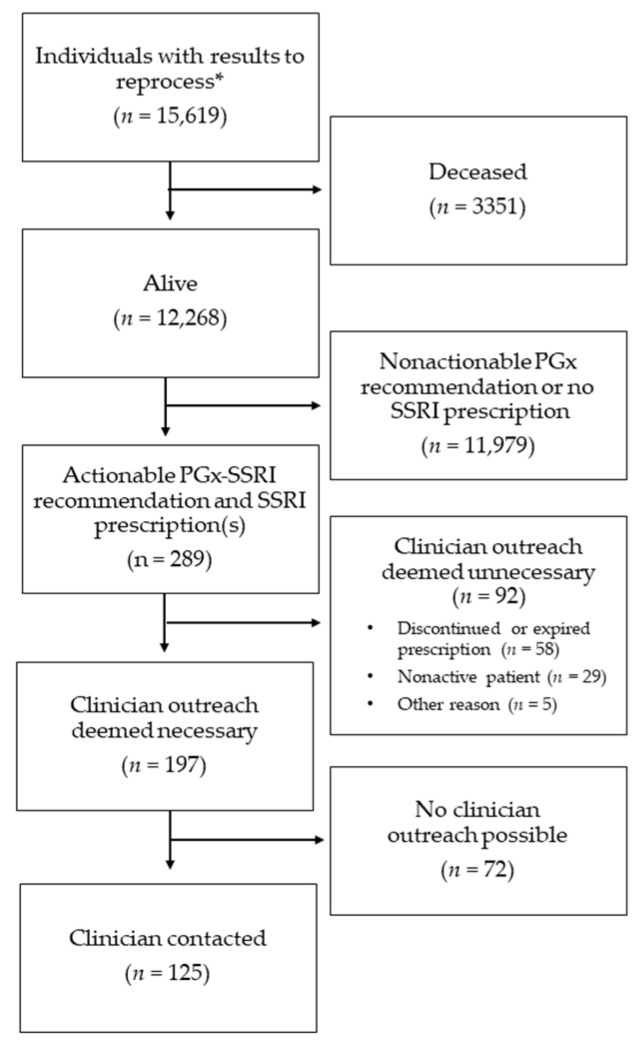
Flow chart of reprocessing initiative. * Reprocessing and reinterpretation included 55 pediatric patients, none of whom were on active SSRI prescriptions.

A total of 15,619 individual patients’ PGx results were reprocessed (Figure 3). The majority of these patients were still alive (78.5%, *n* = 12,268) and aged 18 years or older (99.5%, *n* = 12,213). Of the non-deceased adult patients reprocessed, the median age was 69.5 years old (interquartile range 60.9 to 77.6), 57.5% were male (*n* = 7028), and the majority self-identified as White (84.6%, *n* = 10,338). A total of 21% (*n* = 3278) resulted in CYP2C19 *1/*17 reinterpretations. Among living individuals with prior *CYP2C19* and/or *CYP2D6* results, 289 had an actionable recommendation for SSRI therapy and a prescription for the relevant SSRI medication. After one year, reprocessing resulted in 117 BPAs firing (escitalopram (*n* = 71), citalopram (*n* = 38), and sertraline (*n* = 8)) for reprocessed historic patients. Newly tested patients resulted in 296 SSRI BPA after release of SSRI content. 

### 4.3. Impact

#### 4.3.1. Actionable PGx Interpretations

**Figure 4:**
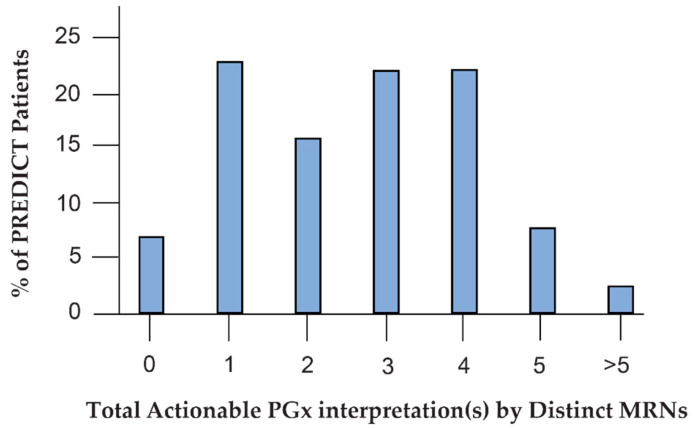
Reprocessing for SSRI content expansion increases drug–gene interaction risk. Thiopurines counted as a drug class instead of separate actionable PGx interpretations. Medical record number (MRN) was used as a surrogate for distinct patient count.

Reprocessing after addition of SSRI CDS revealed a shift in the total number of individuals with actionable PGx results in our patient cohort (Figure 4). A total of 6.9% of patients had no actionable PGx interpretations, 22.9% had 1, 15.9% had 2, 22.1% had 3–4, and 7.7% had 5. Our findings revealed at least one additional PGx risk in 93.21% of the patient cohort. Increase in the number of actionable PGx interpretations is expected as reprocessed historical patients and newly tested patients now have additional SSRI-related DGIs and reinterpretations.

#### 4.3.2. Clinician Contact for New Actionable Recommendations 

After reprocessing, 289 patients were identified as being on an affected SSRI medication through an automated medication scan (Figure 3). Of the patients with SSRI prescriptions, a total of 50.5% (*n* = 146) were for escitalopram, 46.4% (*n* = 134) for citalopram, and 3.1% (*n* = 9) for sertraline. 

For patients with actionable recommendations, 31.8% (*n* = 92) did not necessitate contact of a clinician. The reasons contact was deemed unnecessary included discontinued or expired prescription (20.1%, *n* = 58), nonactive patient (10%, *n* = 29), or recent notes mentioning hospice, moving, patient reported not taking the medication, or completed therapy (1.7%, *n* = 5). To help guide best practices, a total of 43.2% (*n* = 125) of the patient’s clinicians were contacted. For a number of patients, no clinician outreach was possible (25%, *n* = 72) due to challenges identifying an appropriate clinician. The process of manual chart review and initial contact of clinicians took 2 weeks to complete. 

#### 4.3.3. Patient and Provider Notification

One of the unintended consequences of our reprocessing effort was unforeseen automated clinician and patient notifications of “new” laboratory results. We planned to suppress blanket notifications to clinicians and patients and focus on contacting clinicians manually to manage clinical impact. Despite considerable preparatory work, planning, and testing, there were historic linkages across systems that only revealed themselves after the reprocessing was complete, causing patients to receive a message through the patient portal that new results were available. In response, patients contacted their providers, and several of these clinicians contacted the molecular diagnostics lab, PREDICT SMEs, and the PREDICT program staff to understand the situation. Rapid coordination with Health IT partners allowed the release of an orientation message to all clinicians impacted (Figure S2a). For patients, the situation was more complex. The notifications of new results were released into their MHAV portal. Many had not been recently seen at our health care center, and this occurred in the midst of the initial wave of the COVID-19 pandemic. Patients had concerns over their privacy and treatment options related to genetic results. Collaborative efforts were undertaken with Patient Education, the Privacy Office, and the MHAV team to quickly provide explanatory patient outreach and to address additional concerns (Figure S2b). 

#### 4.3.4. Clinical Decision Support

**Figure 5:**
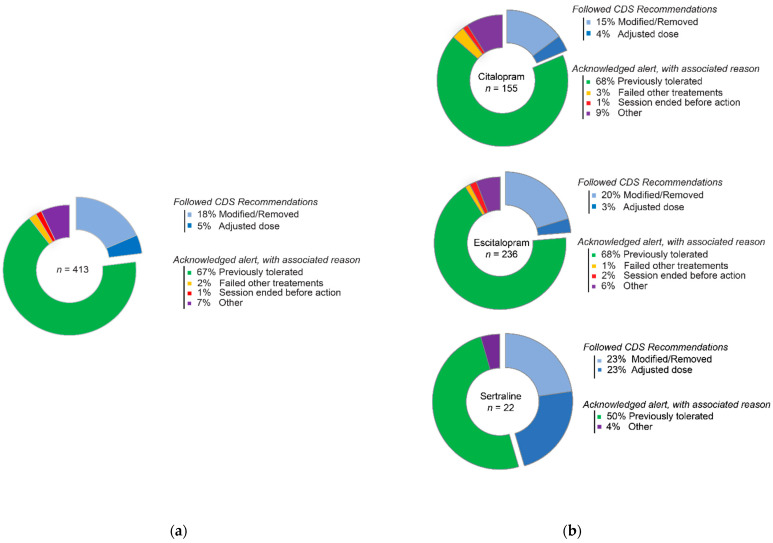
Acceptability and actions taken for SSRI CDS. (**a**) Combined CDS recommendations and acknowledgements for SSRIs. (**b**) Individual escitalopram, citalopram, and sertraline CDS decisions taken by health care providers.

Since the release of the SSRI CDS and reprocessing effort, 413 SSRI BPAs have fired for 160 individual patients involving 259 healthcare providers over a period of one year and four months. The patient population were mainly self-identified as White (90%), male (52%), with a median age of 65 years old (interquartile range 55–73). Age at first BPA encounter was used if multiple BPAs occurred for an individual patient. The BPAs fired in both the inpatient (44.8%, *n* = 185) and outpatient (55.2%, *n* = 228) settings. Escitalopram BPAs were most common (57.1%, *n* = 236), followed by citalopram (37.5%, *n* = 155), and sertraline (5.3%, *n* = 22). Overall, 23% (*n* = 95) of the BPAs resulted in actions aligning with the CDS recommendation including removal of the triggering SSRI order and ordering an alternative agent (18.4%, *n* = 76) or adjusting dose (4.6%, *n* = 19) (Figure 5a). This percentage varied depending on the SSRI, with the lowest percent of CDS recommendation followed for citalopram BPAs (19%) and highest for sertraline BPAs (46%) (Figure 5b). A total of 77% (*n* = 318) of the BPAs resulted in an acknowledgement reason for the following reasons: previously tolerated (66.6%, *n* = 275), failed other treatments (1.9%, *n* = 8), session ended before action (1.5%, *n* = 6), and other (7%, *n* = 29) (Figure 5a).

**Figure 6:**
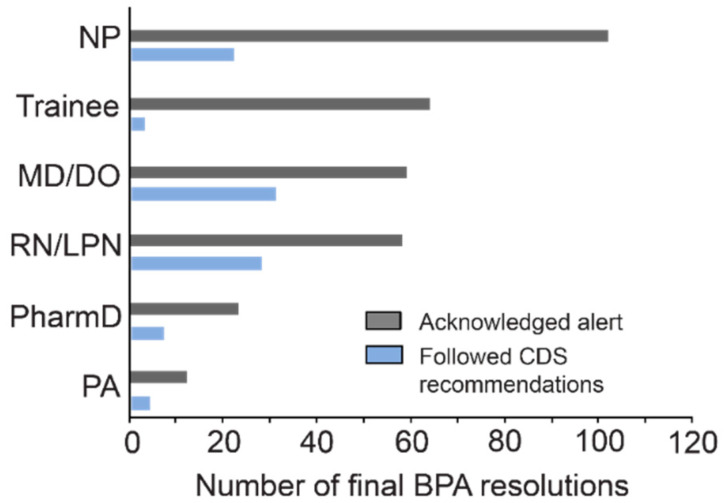
CDS resolution sorted by healthcare provider. Trainees included fellows, physician residents, and medical students. CDS recommendations were followed if provider ordered alternative drug, removed order, or adjusted dose. Acknowledged reasons were previously tolerated, failed other treatments, session ended before action, and other. MD: Doctor of Medicine, DO: Doctor of Osteopathic Medicine, NP: Nurse Practitioner, PA: Physician Assistant.

The healthcare provider that encountered the BPAs most often were nurse practitioners (NP) (30%, *n* = 124), followed by physicians (MD, DO) (21.8%, *n* = 90) (Figure 6). Physicians were the most likely to modify or remove the SSRI order followed by nurses acting as proxies for physicians, physician assistants (PA), pharmacists, nurse practitioners, and physician trainees (34.4%, 32.6%, 25%, 23.3%, 17.7%, 4.5%; respectively). 

## 5. Discussion

### 5.1. Benefits of Reprocessing

Updating of *CYP2C19* interpretations in over 12,000 non-deceased adult patients at our institution resulted in CYP2C19*1/*17 reinterpretations for 21% (*n* = 3278) of individuals. We added SSRI recommendations for all individuals with existing *CYP2C19* and/or *CYP2D6* results, *n* = 289 (2.4%) of whom had actionable recommendations and relevant SSRI prescriptions. Although PGx results are enduring and should last the lifetime of the patient (provided no additional gene variants are required for testing), the interpretations and recommendations are not static. To date, we are unaware of literature discussing reprocessing of historic PGx results. A process for periodic reinterpretation and reprocessing is necessary for PGx results to be efficiently and accurately used by clinicians. Multiple CPIC revisions have been released describing recommendations for antiplatelet drug selection for patients with *CYP2C19* variants since the initial publication in September 2013 [18,19]. Similar revisions have been published for *CYP2D6* variants and opioid drugs, which were initially released in April 2012 and updated in 2020 [16,20,21,22]. Additionally, an array of CPIC drug guidelines use *CYP2C19* and *CYP2D6,* including guidelines for proton pump inhibitors, voriconazole, atomoxetine, and ondansetron, among others, and would be impacted by updates to nomenclature and variant interpretations [23,24,25,26].

Reprocessing PGx results maximizes the clinical utility of a panel test and increases the value of the initial PGx test for patients already tested. Historically tested patients and newly tested patients both received support from the updated SSRI CDS (*n* = 117 and *n* = 296, respectively). Here, we reviewed the methods and findings of our SSRI CDS content expansion, and we believe similar strategies could be leveraged to onboard new CDS, such as atomoxetine and tricyclic antidepressants. Reprocessing historical genetic results for program expansion is a judicious use of institutional resources to parallel the advancement of clinical PGx.

### 5.2. Lessons Learned

Operationally, our reprocessing effort succeeded at communicating with providers the potential concerns related to pharmacogenomic risk re-classification. However, there were unintended consequences that required active management and immediate attention. The automated patient and provider notifications of new “laboratory results” highlights the complexities of reinterpretation when multiple information systems and teams are involved in displaying PGx results across patient- and provider-facing portals. These experiences will inform our future reprocessing plans. More comprehensive communications management is highly advisable. Preemptive messaging via a system-wide alert may be warranted to ensure that clinicians are situationally aware. A targeted explanatory banner within the patient portal system may help provide context and reassurance. Patient engagement and debriefing from the 2020 event have also provided insights as to how to structure outreach and better serve our PREDICT population. 

On the clinical side, one of the main challenges was identifying relevant clinicians that could be contacted to convey changes in interpretations and recommendations. Some patients only engaged specialty clinicians at VUMC with an outside primary care or psychiatry clinician. Although some outside clinicians were able to be contacted within the EHR, we did not go further in contacting clinicians outside of the secure EHR environment. 

### 5.3. Feasibility and Responsibility

The reprocessing course required strategic planning and a multidisciplinary team effort. In addition, the costs and efforts associated with maintenance of a PGx program should not be overlooked. No charges to patients or payors were generated for this reprocessing effort, as reimbursement for reinterpretation-related efforts would be an even newer concept and may not be conceivably recoverable for the foreseeable future. However, we have found that the potential patient impact makes this endeavor a logical pursuit for our program. 

There are also points of contention regarding reinterpretation, such as who to recontact as well as who is responsible for initiating the reprocessing process (e.g., laboratory vs. clinical request vs. program decision). In an ideal scenario, we would contact both clinicians and patients to convey any pertinent reinterpretations and changes in recommendations; however, this is a resource intensive endeavor. Without a clinical relationship and clear understanding of a patient’s medical history, notifying patients of reinterpretations and counseling on changes in clinical recommendations could result in confusion and concerns about privacy. Notifying clinicians, on the other hand, is a more feasible objective to accomplish and may result in more managed, appropriate changes in therapy. In our experience, strong PGx program leadership was necessary to negotiate adequate institutional resources and initiate reprocessing. 

## 6. Conclusions

In summary, reinterpretation and reprocessing of PGx results requires a multidisciplinary team effort and is an important and achievable task. Reprocessing of PGx results creates an impact on patients and the clinicians who care for them. 

Reinterpretation and reprocessing was able to support our programmatic goal of providing enterprise-wide clinician support with up-to-date SSRI CDS for historic and new patients. For future reprocessing efforts, we aim to improve our contact with outside providers, identify a feasible proactive strategy for contacting patients, and ensure that no unintended automated messages are disseminated. As technology advances, we will surely face additional future reprocessing challenges. We will grapple with integration of outside and non-discrete PGx results, extraction of PGx results from Next Generation Sequencing data, and support of PGx results from multiple testing platforms. As PGx results may endure for the lifetime of a patient, continuous effort needs to be made to maintain up-to-date interpretations and recommendations to maximize the full value of PGx testing. Reprocessing will become a key strategy for the maintenance and expansion of PGx CDS. 

## Data Availability

The data presented in this study are not available due to privacy concerns.

## Supplementary Materials

The following are available online at https://www.mdpi.com/article/10.3390/jpm11111051/s1, Figure S1: Example of message sent to clinicians regarding actionable recommendations after reprocessing. Figure S2: Clarification messages sent to providers (a) and patients (b) regarding reprocessing and explanation of the unintended notification.
