# Supplementary figures and images for: Impact of Updating Pharmacogenetic Results: Lessons Learned from the PREDICT Program
Source: J Pers Med. 2021 Oct 20;11(11):1051. doi: 10.3390/jpm11111051 (PMC8617828; doi:10.3390/jpm11111051)

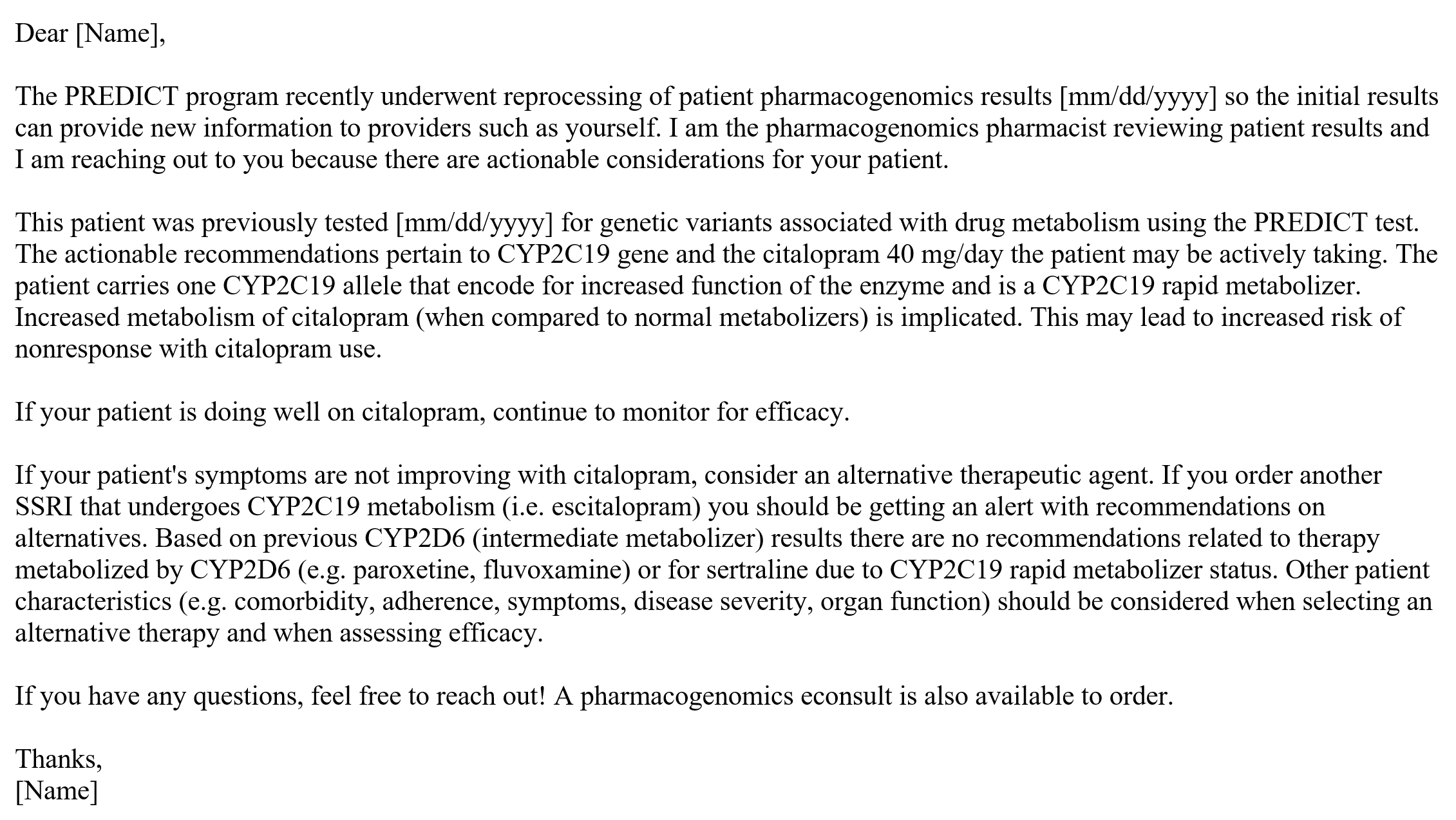

Supplement: Supplementary file 1 [file jpm-11-01051-s001.zip › jpm-1403056-supplementary/Figure S1.PNG]

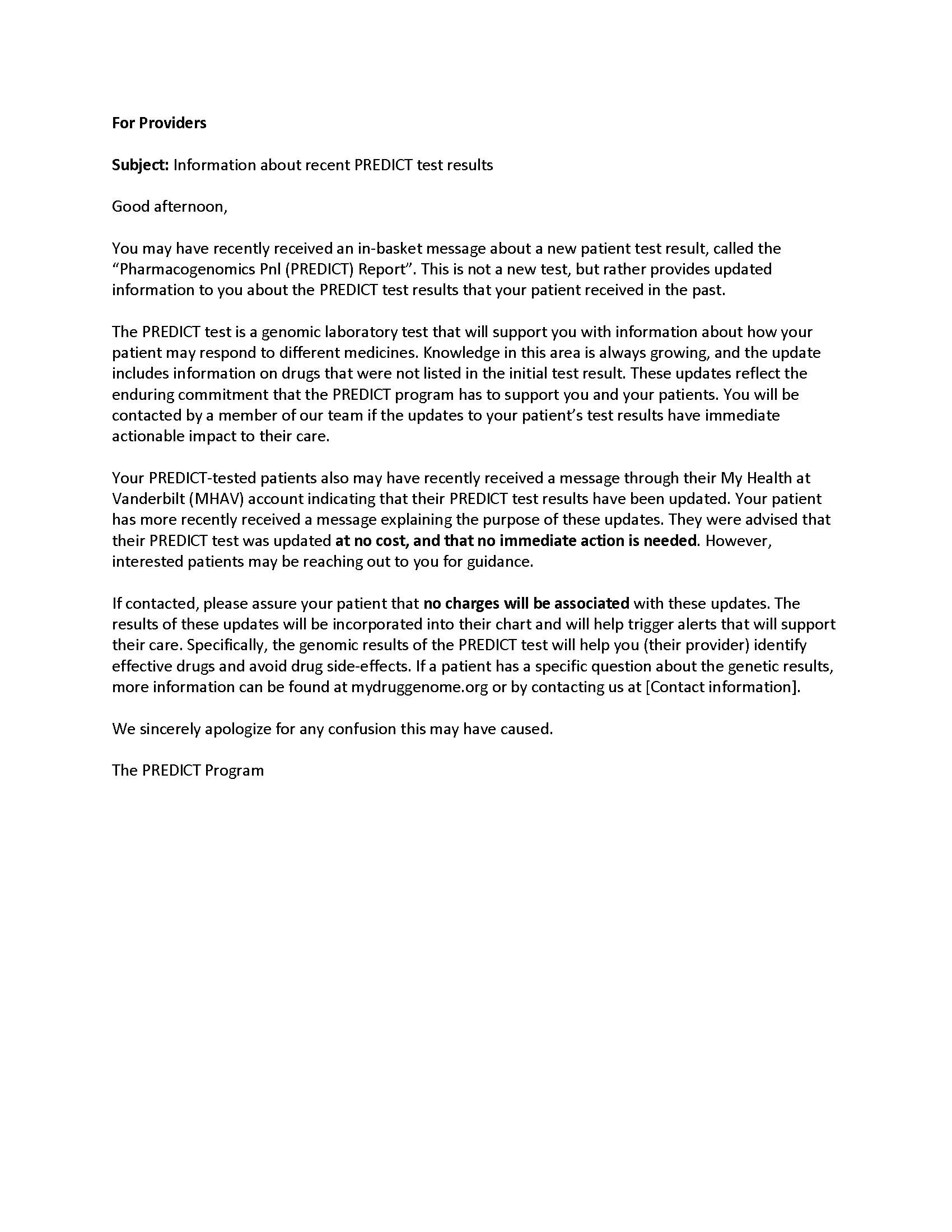

Supplement: Supplementary file 1 [file jpm-11-01051-s001.zip › jpm-1403056-supplementary/Figure S2a.png]

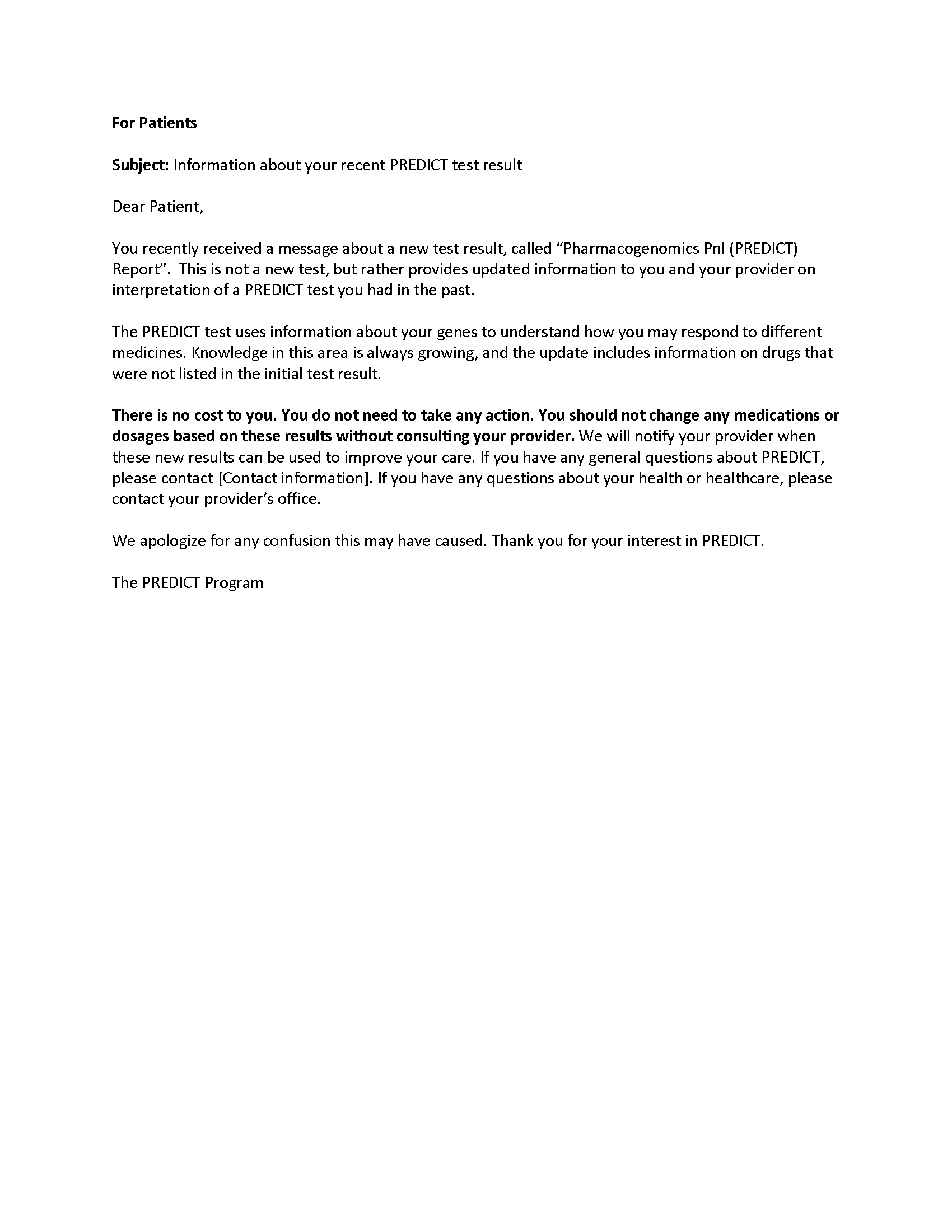

Supplement: Supplementary file 1 [file jpm-11-01051-s001.zip › jpm-1403056-supplementary/Figure S2b.png]
